# Therapeutic potential of human umbilical cord mesenchymal stem cells in the treatment of rheumatoid arthritis

**DOI:** 10.1186/ar3187

**Published:** 2010-11-16

**Authors:** Yanying Liu, Rong Mu, Shiyao Wang, Li Long, Xia Liu, Ru Li, Jian Sun, Jianping Guo, Xiaoping Zhang, Jing Guo, Ping Yu, Chunlei Li, Xiangyuan Liu, Zhenyu Huang, Dapeng Wang, Hu Li, Zhifeng Gu, Bing Liu, Zhanguo Li

**Affiliations:** 1 Department of Rheumatology and Immunology, Peking University People's Hospital, 11 Xizhimen South Street, Beijing, 100044, PR China; 2 Department of Rheumatology and Immunology, Peking University Third Hospital, 49 North Garden Road, Beijing, 100191, PR China; 3 Department of Gynecology and Obstetrics, Peking University People's Hospital, 11 Xizhimen South Street, Beijing, 100044, PR China; 4 Arthritis Clinic and Research Center, Peking University People's Hospital, 11 Xizhimen South Street, Beijing, 100044, PR China; 5 Department of Rheumatology, The Affiliated Hospital of Nantong University, 20 Xi Si Road, Nantong, 226001, PR China; 6 Laboratory of Oncology, Affiliated Hospital of Academy of Military Medical Sciences, 8 Dong Da Street, Beijing, 100071, PR China

## Abstract

**Introduction:**

Rheumatoid arthritis (RA) is a T-cell-mediated systemic autoimmune disease, characterized by synovium inflammation and articular destruction. Bone marrow mesenchymal stem cells (MSCs) could be effective in the treatment of several autoimmune diseases. However, there has been thus far no report on umbilical cord (UC)-MSCs in the treatment of RA. Here, potential immunosuppressive effects of human UC-MSCs in RA were evaluated.

**Methods:**

The effects of UC-MSCs on the responses of fibroblast-like synoviocytes (FLSs) and T cells in RA patients were explored. The possible molecular mechanism mediating this immunosuppressive effect of UC-MSCs was explored by addition of inhibitors to indoleamine 2,3-dioxygenase (IDO), Nitric oxide (NO), prostaglandin E2 (PGE2), transforming growth factor β1 (TGF-β1) and interleukin 10 (IL-10). The therapeutic effects of systemic infusion of human UC-MSCs on collagen-induced arthritis (CIA) in a mouse model were explored.

**Results:**

*In vitro*, UC-MSCs were capable of inhibiting proliferation of FLSs from RA patients, via IL-10, IDO and TGF-β1. Furthermore, the invasive behavior and IL-6 secretion of FLSs were also significantly suppressed. On the other hand, UC-MSCs induced hyporesponsiveness of T cells mediated by PGE2, TGF-β1 and NO and UC-MSCs could promote the expansion of CD4^+ ^Foxp3^+ ^regulatory T cells from RA patients. More importantly, systemic infusion of human UC-MSCs reduced the severity of CIA in a mouse model. Consistently, there were reduced levels of proinflammatory cytokines and chemokines (TNF-α, IL-6 and monocyte chemoattractant protein-1) and increased levels of the anti-inflammatory/regulatory cytokine (IL-10) in sera of UC-MSCs treated mice. Moreover, such treatment shifted Th1/Th2 type responses and induced Tregs in CIA.

**Conclusions:**

In conclusion, human UC-MSCs suppressed the various inflammatory effects of FLSs and T cells of RA *in vitro*, and attenuated the development of CIA *in vivo*, strongly suggesting that UC-MSCs might be a therapeutic strategy in RA. In addition, the immunosuppressive activitiy of UC-MSCs could be prolonged by the participation of Tregs.

## Introduction

Rheumatoid arthritis (RA) is a chronic and systemic disease that primarily attacks synovial joints, leading to articular destruction and functional disability. RA imparts a massive burden on health services worldwide. Efforts to discover new target therapies have achieved considerable success. For instance, TNF-α inhibitors and B-cell-depleting therapies have benefited many RA patients [1,2]. However, these approaches are expensive and none of the currently widely used biological agents reaches longterm drug-free remission [3,4]. Therefore, it is important to develop new and more effective therapy for RA.

In RA, proinflammatory cytokines, such as TNF-α, IL-6, IL-1β and IL-17, play dominant pathological roles. Aberrant T help cells (Th) 17 and Th1 responses have been linked to pathogenesis of RA [5-7]. Furthermore, evidence is accumulating that a defect in number or function of regular T cells (Tregs) is important in the immune imbalance that culminates in RA [8,9]. The fibroblast-like synoviocytes (FLSs) are resident cells of synovial joints, involved in pannus formation, and are key players in the destruction of cartilage and bone in RA joint [10]. The ability of FLSs to stimulate both inflammation and tissue damage suggests that this cell type may be another critical target for the treatment of inflammatory arthritis [11].

Mesenchymal stem cells (MSCs) are cells of stromal origin that can exert profound immunosuppression by modulating T and B cell proliferation and differentiation, dendritic cell maturation and NK activity. These immunoregulatory properties encouraged a possible use of these cells to modulate autoimmune responses and in the treatment of autoimmue diseases [12,13]. To date, the experience of MSCs in the treatment of RA is limited to a few cases, with controversial results from preclinical models [14-18]. As of yet, the most common source of MSCs has been bone marrow. However, aspirating bone marrow is an invasive procedure. In addition, the number and the differentiating potential of bone marrow MSCs (BM-MSCs) decrease with age [19,20]. In contrast, the umbilical cord is a postnatal organ discarded after birth. The collection of umbilical cord MSCs (UC-MSCs) does not require any invasive procedure. In addition to the well-documented self-renewal and multipotent differentiation properties, UC-MSCs possess immunoregulatory capacities that have been permissive to allogeneic transplantation [21]. Given these characteristics, particularly the plasticity and developmental flexibility, the UC-MSCs are now considered an alternative source of stem cells and deserve to be examined in long-term clinical trials [22]. However, very little is known about UC-MSCs, and of note, there has been no report about UC-MSCs in the treatment of RA.

In this study, we reported our findings of the suppressive effect of UC-MSCs on the proliferation, invasive behavior and inflammatory responses of FLSs from RA patients. We also demonstrated that UC-MSCs could inhibit activation of T cells and induced Tregs expression in RA. More importantly, in mice, systemic infusion of UC-MSCs significantly reduced the severity of collagen-induced arthritis (CIA). In addition, the possible mechanism(s) underlying the UC-MSCs-mediated inhibitory effect were explored.

## Materials and methods

### Isolation, culture and differentiation of UC-MSCs

This study was approved by the Research Ethics Committee at the Beijing University People's Hospital (FWA00001384). All participants provided written informed consent. Fresh human umbilical cords (*n *= 5) were obtained after birth and collected in Hanks' Balanced Salt Solution at 4°C. Umbilical arteries and veins were removed, and the remaining tissue was transferred into a sterile container in Minimum Essential Medium (MEM-α) (Invitrogen, Carlsbad, CA, USA) with antibiotics (penicillin 100 IU/ml, streptomycin 100 μg/ml; Invitrogen) and was then dissected into cubes of about 0.5 cm^3 ^and centrifuged at 250 g for five minutes. The explants were transferred to a 25 cm^2 ^flask containing the MEM-α along with 10% fetal bovine serum (Invitrogen). They were left undisturbed for three to four days to allow migration of cells from the explants, at which point the media was replaced. They were re-fed and passaged as necessary. After three passages, the cells were harvested and stained with fluorescein-conjugated monoclonal antibody against CD14, CD45, CD34, HLA-DR, CD44, CD73, CD90 and CD29. (BD Pharmingen, San Diego, CA, USA), followed by analyzing with flow cytometry (FACS Calibur, Becton, Dickinson and Company, Franklin Lakes, NJ, USA). The UC-MSCs were then used directly for culture or stored in liquid nitrogen for later use.

### Osteogenic differentiation

To induce osteogenic differentiation, third- to seventh-passage cells were treated with osteogenic medium for three weeks with medium changes twice weekly. Osteogenesis was assessed at weekly intervals. Osteogenic medium consists of MEM-α supplemented with 0.1 μM dexamethasone (Sigma, St. Louis, MO, USA), 10 mM β-glycerol phosphate (Sigma) and 0.2 mM ascorbic acid (Sigma).

### Adipogenic differentiation

To induce adipogenic differentiation, second- to fifth-passage cells were treated with adipogenic medium for three weeks. Medium changes were carried out twice weekly and adipogenesis was assessed at weekly intervals. Adipogenic medium consists of MEM-α supplemented with 0.5 mM 3-isobutyl-1-methylxanthine (Sigma), 1 μM hydrocortisone (Sigma), 0.1 mM indomethacin (INDO, Sigma) and 10% rabbit serum (Sigma).

### Isolation and culture of FLSs and T cells from RA patients

Synovial tissues were obtained from patients with RA (*n *= 5, females, aged from 30 to 60 years) and traumatic patients without arthritis (*n *= 4) at time of knee replacement surgery. Peripheral blood mononuclear cells (PBMCs) isolated from 10 RA patients (females, aged from 35 to 56 years) by density sedimentation on Ficoll-Hypaque gradients were separated immunomagnetically into T cells by negative selection using the RosetteSep enrichment cocktail according to the manufacturer's instructions (Stem Cell Technologies, Vancouver, BC, Canada). The procedure was approved by the ethical committee at the Beijing University People's Hospital (FWA00001384). All patients gave written informed consent. All RA patients fulfilled the criteria of the American College of Rheumatology for the classification of RA [23]. Isolation and culture of FLSs were described previously [24].

### Proliferation assay

UC-MSCs were all irradiated (30 Gray) before being co-cultured with FLSs or T cells. Each culture was performed in triplicate in 96-well flat-bottom microtitre plates (Corning, New York, NY, USA) in a total volume of 0.2 ml MEM-α supplemented with 10% FBS. UC-MSCs were added to the plates at different ratios to FLSs or T cells with the stimulation of TNF-α (PeproTech Inc, Rocky Hill, NJ, USA; 20 ng/ml) or PHA (Sigma, 2 μg/ml). The group in which FLSs or T cells were cultured alone served as negative controls. The plates were incubated in a humidified atmosphere of 5% CO_2 _at 37 ℃ for five days. UC-MSCs were added on Day 4 (1:1 to FLSs) to the total five-day coculture to explore the effects of UC-MSCs on FLSs at late time point. To evaluate the possible mechanisms of the suppressive effect of UC-MSCs, inhibitors of indoleamine 2,3-dioxygenase (IDO), nitric oxide (NO), prostaglandin E2 (PGE2), transforming growth factor β1 (TGF-β1) and IL-10 (that is, 1-methyl-DL-tryptophan (1-MT) (Sigma), N-nitro-L-arginine methyl ester (L-NAME) (Sigma), INDO, anti-TGF-β1 mAb (R&D, Minneapolis, MN, USA) and anti-IL-10 mAb (R&D)) were added to co-cultures at appropriate concentrations.

To compare the suppressive capacity of CD4^+^CD25^+ ^T cells from CIA mice treated with human UC-MSCs and phosphate buffered saline (PBS), the regulatory T cells (Tregs) were purified from the spleens by magnetic cell sorting using the CD4^+^CD25^+ ^regulatory T-cell isolation kit (Miltenyi Biotec, Bergisch Gladbach, Germany) in accordance with the manufacturer's instructions. Tregs (1 × 10^5 ^cells) from human UC-MSC-treated or untreated mice were added to the CD4^+^CD25^- ^responder T cells (1 × 10^5 ^cells), stimulated with anti-CD3 Ab (BD Pharmingen, 5 μg/ml) and anti-CD28 Ab (BD Pharmingen, 5 μg/ml) for five days.

Eighteen hours before the end of culture, 1 μCi of (^3^H) thymidine (GE Healthcare, Amersham, Buckinghamshire, UK) was added to each well. Cells were harvested onto nitrocellulose, and the radioactivity incorporated was counted in a scintillation counter. The FLSs and T cell proliferation was represented as the incorporated radioactivity in counts per minute (c. p. m.) and the results were expressed as c. p. m. ± S.D. of the mean. All experiments in our study including the following study were performed independently at least three times for each point described.

### Transwell culture

FLSs and T cells were cultivated in the lower chamber of a 6.5 mm or 4.26 mm diameter Transwell plate with a 0.4 μm pore size membrane (Corning). UC-MSCs were seeded onto the Transwell membrane of the inner chamber one to two hours before the beginning of the culture. Control culture did not contain UC-MSCs, or UC-MSCs were added directly to the FLSs or T cells. After three or five days, cytokine production or proliferation of FLSs and T cells was determined. The invasive behavior of FLSs was assayed using the Cytoselect 24-Well Cell Migration and Invasion Assay (Cell Biolabs Inc, San Diego, CA, USA) according to the manufacturer's instructions. Briefly, UC-MSCs (150,000), which were fixed with 1% paraformaldehyde, were distributed to wells with FLSs (150,000), or UC-MSCs (150,000) were added in the lower well of the invasion plate, with FLSs (150,000) alone in the well inserts. Forty-eight hours later, the inserts were stained with the cell stain solution and the OD 560 nm was measured in a plate reader.

### Induction and treatment of CIA

Animal experimental protocols were approved by the Ethics Committee of Beijing University People's Hospital (FWA00001384). DBA/1 mice (six to eight weeks old; SLAC Laboratory Animal Center, Shanghai, China) were injected subcutaneously with 150 μg of bovine type II collagen (CII) (Chondrex, Redmond, WA, USA) emulsified in Freund's complete adjuvant, and then given subcutaneous booster injections with 75 μg of CII in Freund's incomplete adjuvant.

Based on clinical scores, mice were monitored for signs of arthritis onset. Clinical arthritis was scored on a scale of 0 to 3, where 0 = no swelling, 1 = slight swelling and erythema, 2 = pronounced edema, and 3 = joint rigidity. Each limb was graded, and the grades were summed to yield the arthritis score for each animal (maximum possible score 12 per animal) [25].

Treatment was begun after the onset of disease (Day 31), when arthritis had become established (arthritis score ≥1). As previously described [16], mice were injected intraperitoneally each day for five days with phosphate buffered saline (PBS) alone, with 1 × 10^6 ^human UC-MSCs, or with 1 × 10^6 ^human FLSs isolated from traumatic joints respectively. In addition, 1 × 10^6 ^dead human UC-MSCs, which were fixed by 4% paraformaldehyde, were injected intraperitoneally into mice with CIA each day for five days. Animals were sacrificed 62 days after immunization wirh CII and their joints were examined in serial sections.

For evaluation of delayed-type hypersensitivity (DTH) reactivity, CIA mice treated with UC-MSCs or not were intradermally injected with 10 μg CII/10 μl PBS in the right ear and with 10 μl PBS in the left ear. Ear swelling was measured 48 hours later with a spring-loaded micrometer.

### Histologic analysis

Formalin-fixed limbs were decalcified and paraffin-embedded using standard histologic techniques. Serial 4 μm sections were cut and stained with hematoxylin and eosin to examine morphologic features and assess the histologic arthritis score. Histopathologic changes are scored using the following parameters. Sections were analyzed microscopically for the degree of inflammation and for cartilage and bone destruction according to the method reported previously [26], using the following scale: 0 = normal synovium, 1 = synovial membrane hypertrophy and cell infiltrates, 2 = pannus and cartilage erosion, 3 = major erosion of cartilage and subchondral bone, and 4 = loss of joint integrity and ankylosis. Each joint was scored separately by two individuals unaware of the treatment protocol.

To trace the migration of transplanted cells *in vivo*, analysis with mAb against human nuclei (Chemicon International, Temecula, CA, USA) was performed following the manufacturer's instructions to detect UC-MSCs in heart, kidney, spleen and joints of mice treated with UC-MSCs.

### Cytokine quantification

After 72 hours of co-culture with or without TNF-α or PHA stimulation, fresh supernatant was collected. Quantitative analyses of IL-6 production were performed by enzyme-linked immunosorbent assay (ELISA) using commercially available kits (R&D). TNF-α and Matrix metalloproteinase 9 (MMP9) quantification were performed on the Luminex-100 system, and the R&D Fluorokine MAP Human Base Kit A or Human MMP MultiAnalyte Profiling Base Kit (R&D) was used. Supernatants of UC-MSCs, FLSs and T cells that were cultured alone served as controls. Cytokine and chemokine levels in the serum of mice with CIA were determined by sandwich ELISA using capture/biotinylated detection antibodies obtained from BD PharMingen.

### Flow cytometric analysis

Tregs were stained with anti-CD4-FITC. Then, cells were fixed and permeabilized by Fix/Perm buffer (eBioscience, San Diego, CA, USA) and stained for anti-forkhead box P3 (Foxp3)-PE. Mice Th1, Th2 or Th17 cells in spleen were stained for anti-CD4-APC, then washed with FACS buffer (PBS plus 1% BSA) and fixed in PBS containing 2% paraformaldehyde. Subsequently, cells were stained for anti-IFN-γ-PE, anti-IL-4-PE or anti-IL-17-PE in FACS buffer containing 0.1% saponin. An appropriate isotype-matched control antibody was used in all FACS analyses. All antibodies were from BD Pharmingen except anti-Foxp3 (eBioscience). Cells were analyzed on a FACS Calibur flow cytometer using Cell Quest software (Becton, Dickinson and Company).

### Statistical analysis

Data were presented as mean ± S.D. The difference between treatment and control groups was analyzed by Mann-Whitney *U *test. *P *< 0.05 was considered significant.

## Results

### Expansion of UC-MSCs *in vitro*

**Figure 1:**
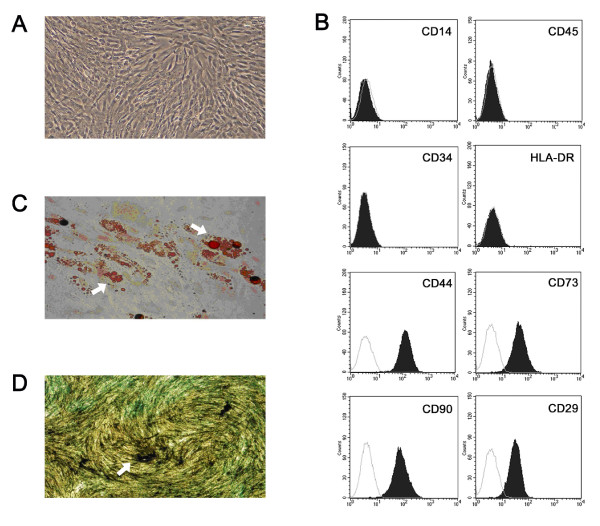
**Characteristics of UC-MSCs**. **(a) **Cell culture of passage 3. Original magnification × 40. The cells had a fibroblast-like morphology. **(b) **Flow cytometric analysis of surface-marker expression on UC-MSCs. They were negative for CD14, CD45, CD34 and HLA-DR, but positive for CD44, CD73, CD90 and CD29. The dotted line is the isotype control. **(c) **Oil red O staining of UC-MSCs after the induction of adipogenic differentiation for 21 days. Arrows indicate lipid roplets. Original magnification × 40. **(d) **Osteogenic differentiation of UC-MSCs staining for alkaline phosphatase. Arrows indicate the accumulation of intracytoplasmic alkaline phosphatase of osteoblast. Original magnification × 40.

The UC-MSCs were successfully isolated and expanded from all the umbilical cords. They had a fibroblast-like morphology, uniformly negative for CD14, CD45, CD34 and HLA-DR, but positive for CD44, CD73, CD90 and CD29 (Figure 1a, b). Functionally, they were capable of differentiating into adipocytes and osteocytes (Figure 1c, d).

### UC-MSCs inhibited proliferation of FLSs from RA patients

**Figure 2:**
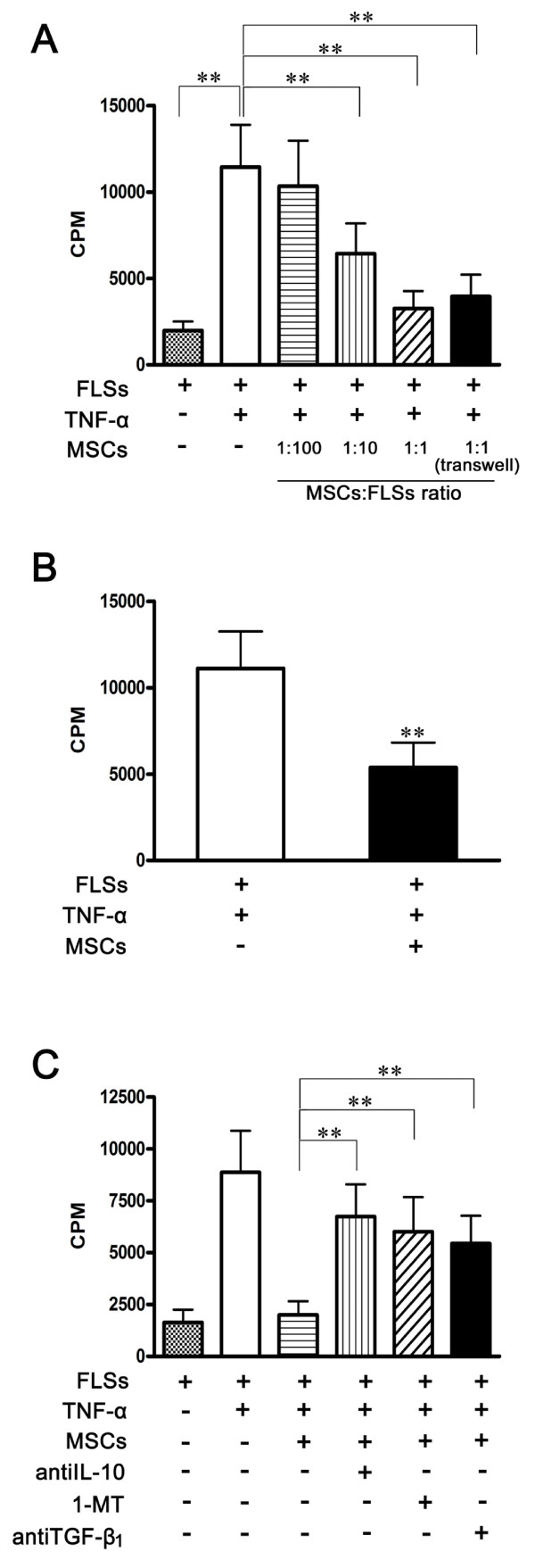
**Effects of UC-MSCs on FLSs proliferation**. **(a) **Compared with the control, TNF-α (20 ng/ml) significantly induced the proliferation of FLSs after five days of culture. UC-MSCs inhibited TNF-α-stimulated-FLSs proliferation in a dose-dependent fashion in the cell-to-cell contact system and also the transwell system. All the data are expressed as the mean ± SD of more than three independent experiments. **P < 0.01 *vs. *the controls. **(b) **FLSs proliferation was significantly inhibited when UC-MSCs were added on the fourth day after the initiation of stimulation in the five-day coculture experiment. All the data are expressed as the mean ± SD of more than three independent experiments. **P < 0.01 *vs. *the control. **(c) **Anti-IL-10, 1-MT and anti-TGF-β1 restored FLSs proliferation. FLSs (1 × 10^4^) were activated with TNF-α in the presence or absence of irradiated MSCs (1 × 10^4^) in 96-well plates. Anti-IL-10 (10 μg/Ml), 1-MT (1 mM) and TGF-β1 antibody (10 μg/mL) were added for five days. The incorporation of (^3^H)-thymidine is shown by CPM. All the data are expressed as the mean ± SD of more than three independent experiments. **P < 0.01.

The FLSs are resident cells of synovial joints, recognized to play an important role in inflammation and joint destruction of RA. Therefore, we attempted to determine the effects of UC-MSCs on the FLSs derived from RA patients. The FLSs isolated from RA patients responded positively to TNF-α (20 ng/ml) when compared with control (11,440 ± 2,452 *vs. *1,985 ± 516, *P *= 0.000). The UC-MSCs significantly inhibited the proliferation of TNF-α-stimulated-FLSs in the cell-to-cell contact and the transwell system, and the effect was dose dependent (Figure 2a). Moreover, such inhibitory effects were profound even when UC-MSCs were added on the fourth day in an experiment of five-day coculture (11,110 ± 2,142 *vs. *5,379 ± 1,435, *P *= 0.000, Figure 2b).

### Soluble factors involved in the suppressive effect of UC-MSCs on the proliferation of FLSs from RA patients

Since IDO, NO, PGE2, IL-10 and TGF-β1 are key factors in MSCs-mediated inhibition [27-30], co-culture experiments were performed using the corresponding inhibitors. They included 1-MT (1 mM), an inhibitor of IDO enzymatic activity, INDO (5 μM), an inhibitor of PGE2 synthesis, L-NAME (1 mM), a specific inhibitor of NO synthase, anti-TGF-β1 monoclonal antibody (10 μg/ml) and anti-IL-10 monoclonal antibody (10 μg/ml). As shown in Figure 2c, TNF-α-mediated FLSs proliferation could be sufficiently restored by anti-IL-10, 1-MT and anti-TGF-β1, respectively, suggesting that those soluble factors were the key mediators in UC-MSCs-mediated inhibition. However, IDO and PGE2 were not found involved in the suppression of UC-MSCs on FLSs (data not shown).

### UC-MSCs suppressed the invasive behavior and MMP9 expression of FLSs from RA patients

**Figure 3:**
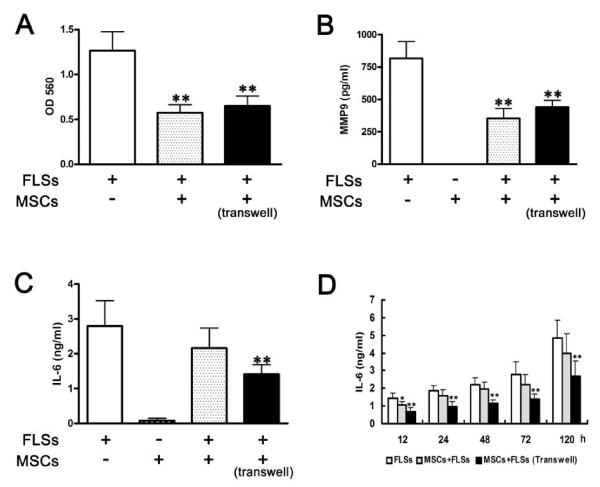
**Effects of UC-MSCs on the invasive behavior and IL-6 production of FLSs *in vitro***. **(a) **Invasive behavior of FLSs in Matrigel matrix was measured in the transwell system. Forty-eight hours after seeding on matrix the number of FLSs grown through Matrigel and transwell membrane was detected. All the data are expressed as the mean ± SD of more than three independent experiments. ** *P *< 0.01 *vs. *the control. **(b) **FLSs (2 × 10^4^) from RA patients and UC-MSCs (2 × 10^4^) were separated in the transwell system or cocultured in the cell-to-cell contact system in 24-well plates. After 72 hours, MMP9 in culture supernatants were determined. MMP9 production was inhibited both in the cell-to-cell contact system and the transwell system. All the data are expressed as the mean ± SD of more than three independent experiments. **P < 0.01, *vs. *FLSs alone. **(c) **FLSs (2 × 10^4^) from RA patients and UC-MSCs (2 × 10^4^) were separated in the transwell system or cocultured in the cell-to-cell contact system in 24-well plates. After 72 hours, IL-6 in culture supernatants was determined. IL-6 production was inhibited in the transwell system. All the data are expressed as the mean ± SD of more than three independent experiments. **P < 0.01 *vs. *FLSs alone. **(d) **Time course of IL-6 production. At different time points, IL-6 was downregulated only in the transwell system. **P < 0.01, *P < 0.05 *vs. *FLSs alone, respectively. All the data are expressed as the mean ± SD of more than three independent experiments.

The invasive property of RA patients-derived FLSs has been shown to correlate with the disease severity and radiographic damage [31]. The MMPs are key mediators of the invasive phenotype of FLSs [32]. Therefore, we further investigated the effect of UC-MSCs on the invasive behavior of FLSs by the Cell Migration/Invasion Assay, and the MMP9 secretion of FLSs. As a result, the invasive behavior of FLSs was significantly inhibited when they were co-cultured with UC-MSCs in the cell-to-cell contact (1.27 ± 0.21 *vs. *0.57 ± 0.09) and the transwell system (1.27 ± 0.21 *vs. *0.65 ± 0.11), (Figure 3a). Consistently, the production of MMP9 was significantly downregulated by co-culture with UC-MSCs in both systems (Figure 3b).

### UC-MSCs suppressed the inflammatory response of FLSs from RA patients

FLSs from RA patients confer both inflammation and tissue damage. One of the critical mediators of inflammation in RA is the proinflammatory cytokine IL-6. Interestingly, the UC-MSCs could downregulate the IL-6 production in the transwell but not the cell-to-cell contact system (Figure 3c) both in single time point and in dynamic study (Figure 3d).

### UC-MSCs induced hyporesponsiveness of T lymphocytes from RA patients

**Figure 4:**
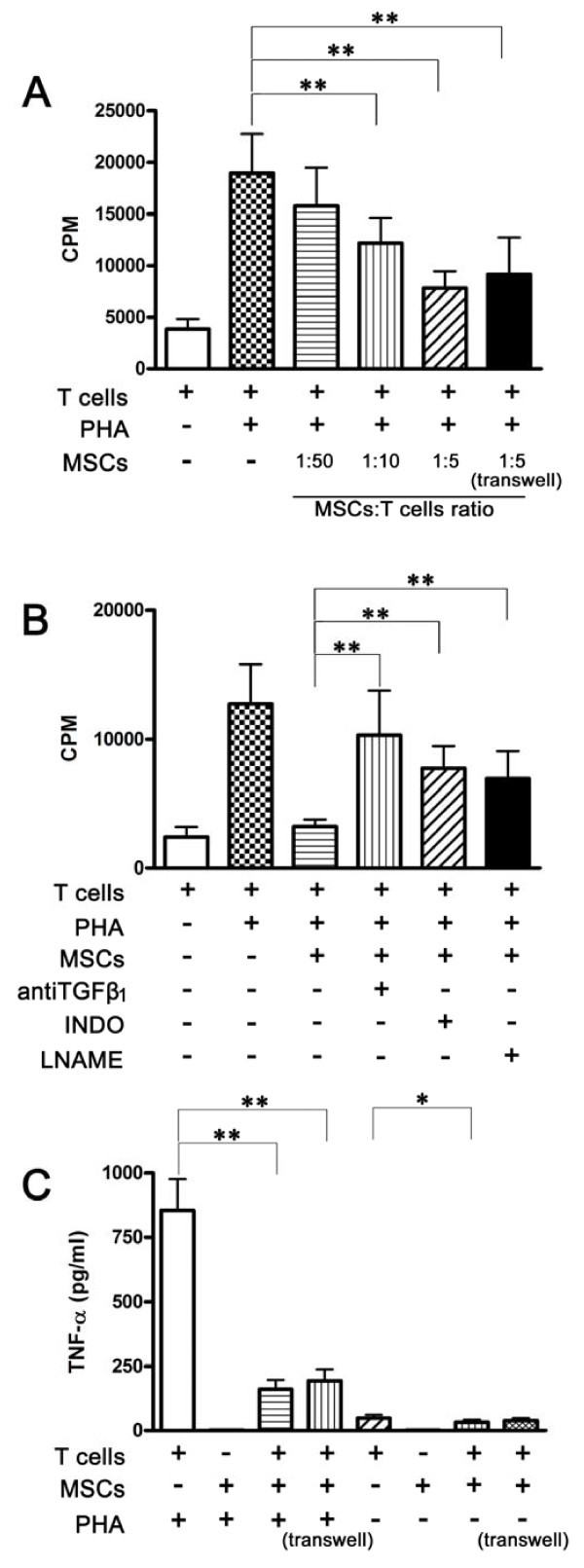
**Effects of UC-MSCs on T cell proliferation and cytokine production**. **(a) **UC-MSCs inhibited PHA-induced T-cell proliferation in a dose-dependent fashion. T cells (1 × 10^5^) were activated with PHA in the presence or absence of irradiated UC-MSCs in different ratio in 96-well plates. Inhibition of T cell proliferation was also found in the transwell system. All the data are expressed as the mean ± SD of more than three independent experiments. **P < 0.01, *vs. *the control. **(b) **Anti-TGF-β1, INDO and L-NAME restored T-cell proliferation. T cells (1 × 10^5^) were activated with PHA in the presence or absence of irradiated UC-MSCs (2 × 10^4^) in 96-well plates. The incorporation of (^3^H)-thymidine is shown by CPM. All the data are expressed as the mean ± SD of more than three independent experiments. **P < 0.01. **(c) **UC-MSCs suppressed T cells from producing pro-inflammatory cytokine TNF-α. T cells (1 × 10^6^) from RA patients and UC-MSCs (5 × 10^4^) were separated in the transwell system or cocultured in the cell-to-cell contact system in 24-well plates. After 72 hours, TNF-α in culture supernatants was determined. All the data are expressed as the mean ± SD of more than three independent experiments. ** *P *< 0.01, * *P *< 0.05 *vs. *the controls, respectively.

Several studies have shown that BM-MSCs could induce hyporesponsiveness of T lymphocytes. However, such investigations are limited for UC-MSCs, particularly so far no studies have been done in RA. As shown in Figure 4a, proliferation of T lymphocytes from RA patients was significantly suppressed by UC-MSCs with a dose-dependent manner, regardless in the cell-to-cell contact or the transwell system. Subsequently, we tried to determine which soluble factors involved in the suppressive process. As shown in Figure 4b, the suppressive effect of UC-MSCs on T cells mainly depended on TGF-β1 (*P *= 0.000), PGE2 (*P *= 0.000) and NO (*P *= 0.000).

In RA pathogenesis, TNF-α plays a central role in the pro-inflammatory cytokine cascade [33]. We then asked whether UC-MSCs-mediated hyporesponsiveness of T cells was associated with TNF-α production. As a result, we observed that UC-MSCs potently decreased the production of TNF-α, both in the cell-to-cell contact and the transwell system, especially in PHA activated T cells (Figure 4c).

### UC-MSCs induced Tregs from RA patients

**Figure 5:**
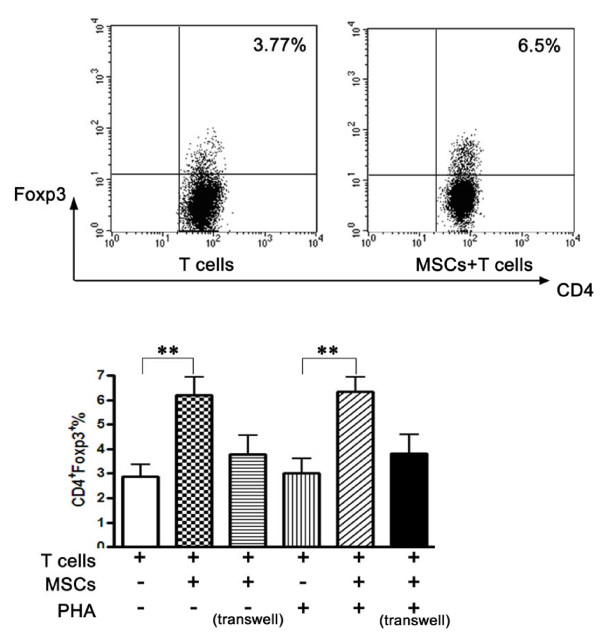
**UC-MSCs induced regulatory T cells expansion**. T cells (1 × 10^6^) isolated from RA patients were cocultured with UC-MSCs (5 × 10^4^) in the absence or presence of PHA in 24-well plates. After three days, regulatory T cells expression was analyzed in the CD4^+ ^T cell fraction by flow cytometry. Numbers represent the mean percentage of positive cells from different groups. All the data are expressed as mean c.p.m. ± S.D, * *P *< 0.05.

Given the concept that Tregs play a critical role in the maintenance of self-immune tolerance in RA [34], UC-MSCs exert an immunoregulatory function on FLSs and T cells. The next intriguing question is whether UC-MSCs play a role in the induction of Tregs in RA. Recent studies demonstrated that not all CD4^+^CD25^bright ^cells coexpressed Foxp3, while some Foxp3^+ ^cells resided in the CD25^dim ^or CD25^- ^population [35]. In this study, the expression of FoxP3 on CD4^+ ^T cells (CD4^+^Foxp3^+^) was defined as Tregs. Notably, the percentages of CD4^+ ^Foxp3^+ ^T cells were significantly higher in the presence of UC-MSCs, irrespective of PHA stimulation (Figure 5).

### UC-MSCs prevented tissue damage in CIA

**Figure 6:**
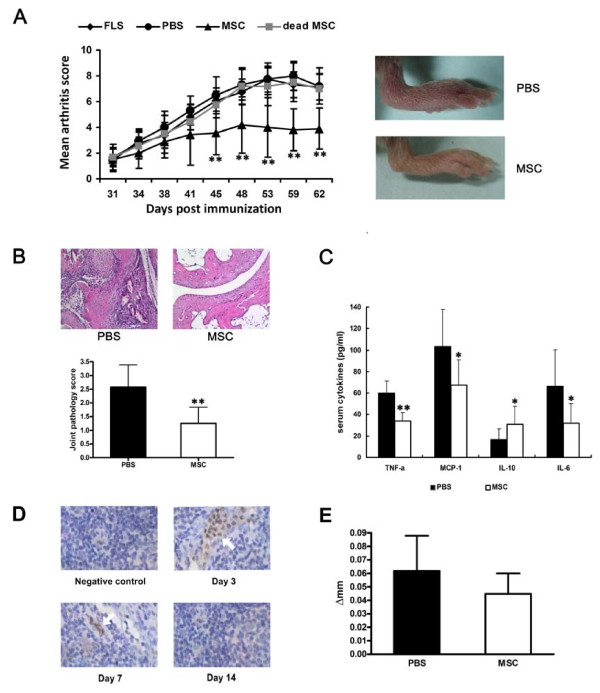
**UC-MSCs prevented tissue damage and inflammatory responses in CIA**. **(a) **Treatment was begun after the onset of disease (arthritis score≥1). PBS and PBS containing 1 × 10^6 ^UC-MSCs were injected intraperitoneally each day for five days to mice with CIA. The severity of CIA was progressively attenuated in UC-MSCs treated mice, as compared with PBS treated mice. *N *= 10, **P < 0.01, *vs. *the PBS controls. All the data are expressed as the mean ± SD. **(b) **H & E-stained sagittal sections of joints from CIA mice. PBS treated mice showed a marked mononuclear cell infiltration, severe synovitis, pannus formation and bone erosion. However, the majority of joints from mice injected with UC-MSCs had normal morphology with a smooth articulation cartilage surface, and an absence of inflammatory cell infiltrate and pannus formation. Original magnification × 100. *N *= 10, **P < 0.01, *vs. *the PBS controls. All the data are expressed as the mean ± SD. **(c) **UC-MSCs treatment reduced inflammatory responses in CIA. There were reduced levels of proinflammatory cytokines and chemokines (TNF-α, IL-6 and MCP-1) and increased levels of the anti-inflammatory/regulatory cytokine (IL-10) in sera of UC-MSC-treated mice, in comparison with PBS treated mice. *N *= 10, ** *P *< 0.01, * *P *< 0.05, respectively. All the data are expressed as the mean ± SD. **(d) **UC-MSCs were detected in the spleen of CIA mice. mAb against human nuclei was used to detect human UC-MSCs in CIA mice, on Day 3 and Day 7, UC-MSCs were detected in the spleen. Arrows indicate human UC-MSCs in the spleen. Original magnification × 200. **(e) **DTH responses in UC-MSC-treated or untreated CII immunized mice. Values are the mean ± SD. *N *= 5.

The immunosuppressive effects of UC-MSCs on T cells and FLSs in human RA promoted us to investigate the potential therapeutic effects of UC-MSCs in CIA, which is an arthritis model that shares a number of clinical, histologic and immunologic features of RA. As shown in Figure 6a, the severity of CIA was progressively attenuated in UC-MSCs treated mice, as compared with PBS treated mice. Moreover, the therapeutic effect was specific to viable human UC-MSCs, because dead human UC-MSCs and human FLSs from traumatic patients without arthritis failed to prevent the progression of arthritis. The therapeutic effects of UC-MSCs on CIA in mice were further verified by histological examination at the endpoint of clinical study. We observed that control mice exhibited a marked mononuclear cell infiltration, severe synovitis, pannus formation and bone erosion. In contrast, the majority of joints from mice injected with UC-MSCs had normal morphology with a smooth articulation cartilage surface, and an absence of inflammatory cell infiltrate and pannus formation (Figure 6b).

### UC-MSCs treatment reduced inflammatory responses in CIA

The clinical amelioration and histological verification in CIA in mice strongly suggests that UC-MSCs are a potent tolerogenic agent that could suppress the autoimmune responses in CIA. We next investigated the effect of UC-MSCs on production of inflammatory mediators that are mechanistically linked to CIA. As shown in Figure 6c, human UC-MSCs injection significantly downregulated protein expression of various proinflammatory cytokines and chemokines (TNF-α, IL-6 and monocyte chemoattractant protein-1 (MCP-1)), as well as upregulted the anti-inflammatory/regulatory cytokine (IL-10).

### UC-MSCs were detected in the spleen of CIA mice

We traced the UC-MSCs in the recipient organism by the detection of mAb against human nuclei in heart, kidney, spleen and joints of mice treated with UC-MSCs. As a result, human UC-MSCs were not detectable by immunohistochemistry in the joints of UC-MSC-treated mice, suggesting that injected UC-MSCs did not restore tissue integrity by mechanisms of tissue repair (data not shown). However, we were able to detect these cells at intermediate time points during the course of the disease in spleen (Figure 6d), but not in other organs, which suggested that UC-MSCs possibly circulate through the bloodstream after the transfusion, after Day 7, human UC-MSCs were negative in the spleen.

### Lymphocyte priming was not affected by UC-MSCs

DTH responses, as evident from the data shown in Figure 6e, suggested that UC-MSCs did not affect priming of antigen-specific T lymphocytes. Because the DTH response was positively recalled using murine CII in mice in all experimental groups, and no statistically significant differences between groups were observed, albeit the response tended to be less vigorous in MSC-treated mice.

### UC-MSCs treatment shifted Th1 toward Th2 and induced Tregs in CIA

**Figure 7:**
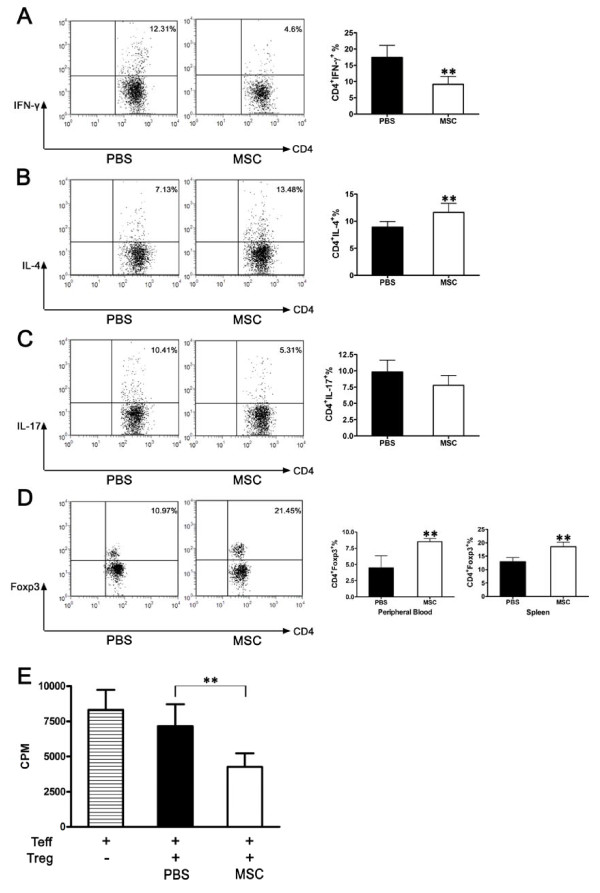
**Effects of UC-MSCs on T cell subtypes in CIA model**. **(a) **UC-MSCs downregulated Th1-type response. UC-MSCs decreased the number of IFNγ-producing Th1 cells, *n *= 6, **P < 0.01. All the data are expressed as the mean ± SD. **(b) **UC-MSCs upregulated Th2-type response. UC-MSCs increased the number of IL4-producing Th2 cells. *N *= 6, **P < 0.01. All the data are expressed as the mean ± SD. **(c) **UC-MSCs tended to decrease Th17-type response. UC-MSCs tend to decrease the number of IL-17-producing Th17 cells. *N *= 6. All the data are expressed as the mean ± SD. **(d) **UC-MSCs treatment induced Tregs in CIA. Percentages of CD4^+^FoxP3^+ ^cells in spleen and peripheral blood in UC-MSCs treated group were higher than the PBS control group. *N *= 6, **P < 0.01. All the data are expressed as the mean ± SD. **(e) **As compared with Tregs isolated from PBS-treated mice, CD4^+^CD25^+ ^T cells isolated from UC-MSC-treated mice functioned as suppressive Tregs, since they inhibited the proliferation of effective T cells (Teff). *N *= 6, **P < 0.01. All the data are expressed as the mean ± SD.

Initially, CIA was considered to be a Th1-mediated disease; however, recent studies have revealed that another T cell subset, -Th17 cells, is also pathogenic in CIA [5,6]. It raises the possibility that the interventions targeting both the IFN-γ (Th1) and the IL-17 (Th17) axes might be more promising therapeutic approaches for CIA [36]. By analyzing the intracellular cytokine expression in the spleen CD4^+ ^T cells, we demonstrated that UC-MSCs could downregulate IFN-γ-producing Th1 cells (Figure 7a) and tend to decrease IL-17-producing Th17 cells (Figure 7c), while upregulated IL-4-producing Th2 cells (Figure 7b).

Several studies have shown that IL-10 producing Tregs confer significant protection against CIA by inhibiting the activation of autoreactive Th1 cells [37,38]. Downregulation of the inflammatory Th1 and the elevated IL-10 levels by UC-MSCs prompted us to further investigate the effect of Tregs in immunosuppressant action of UC-MSCs *in vivo*. As shown in Figure 7d, we found that there were significantly higher numbers of CD4^+^Foxp3^+ ^Tregs in spleen and peripheral blood in the UC-MSC-treated mice than the PBS treated mice. Moreover, CD4^+^CD25^+^T cells isolated from human UC-MSC-treated mice functioned as suppressive Treg cells, since they inhibited the proliferation of syngeneic T cells stimulated with CD3 and CD28 (Figure 7e).

## Discussion

In the present study, we provided evidence that UC-MSCs can exert a profound inhibitory effect on FLSs and T cells from RA patients. They could suppress proliferation, the invasive behavior and inflammatory responses of FLSs, inhibit activation of T cells and induce the Tregs expression. Furthermore, we showed that UC-MSC mediated suppression on T cells and FLSs proliferation through several soluble factors, including IDO, PGE2, NO, IL-10 and TGF-β1, respectively. Systemic infusion of UC-MSCs significantly reduced the severity of CIA in mice. The improvement of clinical manifestation was accompanied by the decreased secretion of various inflammatory cytokines and chemokines, and the downregulated Th1/Th17 cells. Furthermore, in the UC-MSCs treated mice, the expansion of Th2/Tregs and the production of anti-inflammatory IL-10 were elevated.

MSCs have the capability of self-renewal and differentiation into various lineages of mesenchymal tissues. Moreover, MSCs have been consistently shown to exert a potent immunosuppressive effect superior in magnitude to any other immunosuppressive cell types thus far described [39]. Compared with those from bone marrow, MSCs derived from UC have higher proliferative potency, stronger differentiation capacity, and lower risk for viral contamination. However, their therapeutic potential in the treatment of RA has not been investigated.

Recently, the FLSs have been shown to straddle both components of RA, the immune activation and tissue destruction. Therefore, targeting FLSs may abrogate the disease progression [40]. Our data demonstrated that UC-MSCs could inhibit the proliferation of TNF-α stimulated FLSs. Notably, delayed addition of UC-MSCs maintained such inhibitory effects, suggesting that the transplantation of these cells is practicable and effective for treatment of RA. Interestingly, the invasive behavior of FLSs was inhibited by UC-MSCs, indicating that UC-MSCs might be potentially important in the inhibition of bone erosion in RA.

T cells are believed to play a critical role in orchestrating the inflammatory response in RA. Suppression of T cell responses is of great importance in RA treatment, as evidenced by the facts that allogeneic BM-MSCs and hASCs both suppress the responses of CII-reactive T cells in RA [17,18]. In agreement, we observed that UC-MSCs could inhibit the PHA-stimulated-T cell proliferation and secretion of TNF-α. Similar to RA, Th1 and Th17 cell-mediated responses play an important role in the pathogenesis of CIA [41]. Our results demonstrated that administration of human UC-MSCs could downregulate IFN-γ-producing Th1 cells and tend to decrease IL-17-producing Th17 cells, while upregulate IL-4-producing Th2 cells in mice CIA. Tregs play an important role in the prevention of autoimmunity, and it has been demonstrated that they could modulate the severity of CIA [37,42]. Several studies have shown that BM-MSCs and hASCs could recruit, regulate and maintain the T-regulatory phenotype and function over time [43]. In this study, we found UC-MSCs could also induce the Tregs, both *in vitro *and *in vivo*, suggesting that the immunosuppressive activity of UC-MSCs could be prolonged by the participation of Tregs. However, the observation that the DTH response to the immunizing antigen existed in UC-MSC-treated mice indicates that priming of T lymphocytes occurred. Therefore, maybe a complex mechanism existed in the suppressive effect of UC-MSCs.

To date, the molecular mechanisms responsible for the immunosuppressive effects of MSCs have not been completely understood. In BM-MSCs, there have been no agreements among different research groups. However, the main focus is on the soluble factors including IDO, NO, PGE2, IL-10 and TGF-β1 [27-30]. A recent study identified TGFβ1 as a critical mediator involved in the suppressive response of human BM-MSCs on CII-activated PBMCs from RA patients [17]. However, the TGFβ1 blockade did not significantly affect the immunosuppressive action of hASCs on T cells from RA patients [18], suggesting that MSCs of different origins maybe mediated suppression through different cytokines. In this study, we demonstrated that TGF-β1, PGE2 and NO are potent modulators involved in UC-MSCs mediated T-cell inhibition, while IDO, TGF-β1 and IL-10 were mainly involved in the suppressive effect of UC-MSCs on FLSs.

Systemic administration of human UC-MSCs in established CIA in mice significantly ameliorated the clinical and histopathologic severity of the disease. The therapeutic effect was xenogeneic, which means that the immunosuppressive action of UC-MSCs is major histocompatibility complex unrestricted and that the infused UC-MSCs are sufficiently well immunotolerated by the host.

Direct evidence of the beneficial effect is that administration of UC-MSCs attenuated systemic inflammation in CIA in mice. UC-MSCs downregulated the production of the proinflammatory cytokines TNF-α, and IL-6 *in vitro *and *in vivo*. In addition, MCP-1 is a member of the CC family and could be induced by inflammatory cytokines. Several groups have detected MCP-1 in the synovial fluid of RA patients, with markedly higher concentrations than those in other rheumatic diseases, including osteoarthritis [44]. Therefore, reduction of MCP-1 could partly explain the absence of inflammatory infiltrates in the synovium of mice treated with human UC-MSCs. Moreover, UC-MSCs increased the levels of the antiinflammatory cytokine IL-10. Aside from its role as an antiinflammatory factor [45], IL-10 is a signature cytokine for Tregs, and plays a key role in the control of self-antigen-reactive T cells *in vivo *[38]. The upregulation of IL-10 is in line with the induction of Tregs *in vitro *and *in vivo *in our study. Moreover, in our experiments we did not find UC-MSCs in the joint of the CIA mice. The short-term presence of UC-MSCs in the spleen suggests that the therapeutic effect of UC-MSCs does not rely on the capacity to engraft and survive long-term in the appropriate target organs. More likely, UC-MSCs could "educate" other cells to inhibit the pathogenic immune reaction.

## Conclusions

In this study, UC-MSCs exerted a profound inhibitory effect on the proliferation, invasive behavior and inflammatory responses of FLSs, suppressed T cell activation and induced the generation of Tregs. Most importantly, cell-based therapy using human UC-MSCs significantly ameliorated CIA in mice. These data suggest that UC-MSCs might be therapeutic perspectives in RA.

## Abbreviations

1-MT: 1-methyl-DL-tryptophan; BM-MSC: bone marrow MSC; CII: type II collagen; CIA: collagen-induced arthritis; c. p. m.: counts per minute; DTH: delayed-type hypersensitivity; ELISA: enzyme-linked immunosorbent assay; FLS: fibroblast-like synoviocyte; FOXP3: forkhead box P3; IL-10: interleukin 10; IDO: indoleamine 2,3-dioxygenase; INDO: indomethacin; L-NAME: N-nitro-L-arginine methyl ester; MCP: monocyte chemoattractant protein; MSC: mesenchymal stem cell; NO: nitric oxide; MEM-α: Minimum Essential Medium; MMP9: matrix metalloproteinase 9; PBMCs: peripheral blood mononuclear cells; PGE2: prostaglandin E2; PHA: phytohemagglutinin; RA: rheumatoid arthritis; TH: T help cells; TGF-β1: transforming growth factorβ1; TREGS: regular T cells; UC-MSC: umbilical cord MSC.

## Competing interests

The authors declare that they have no competing interests.

## Authors' contributions

ZGL directed the research. YYL and RM designed the research, performed the experiments, analyzed and interpreted data and drafted the manuscript. SYW, LL, RL, XL and JS collected, analyzed and interpreted the data. JPG and BL analyzed, interpreted data and revised the manuscript. XPZ, JG, PY, CLL, XYL, ZYH, DPW, HL and ZFG collected data.
